# Train Distance Estimation for Virtual Coupling Based on Monocular Vision

**DOI:** 10.3390/s24041179

**Published:** 2024-02-11

**Authors:** Yang Hao, Tao Tang, Chunhai Gao

**Affiliations:** 1 School of Electronic and Information Engineering, Beijing Jiaotong University, Beijing 100044, China; ttang@bjtu.edu.cn; 2 Traffic Control Technology Co., Ltd., Beijing 100070, China; chunhai.gao@bj-tct.com

**Keywords:** urban rail transit, autonomous driving, object detection, monocular vision

## Abstract

By precisely controlling the distance between two train sets, virtual coupling (VC) enables flexible coupling and decoupling in urban rail transit. However, relying on train-to-train communication for obtaining the train distance can pose a safety risk in case of communication malfunctions. In this paper, a distance-estimation framework based on monocular vision is proposed. First, key structure features of the target train are extracted by an object-detection neural network, whose strategies include an additional detection head in the feature pyramid, labeling of object neighbor areas, and semantic filtering, which are utilized to improve the detection performance for small objects. Then, an optimization process based on multiple key structure features is implemented to estimate the distance between the two train sets in VC. For the validation and evaluation of the proposed framework, experiments were implemented on Beijing Subway Line 11. The results show that for train sets with distances between 20 m and 100 m, the proposed framework can achieve a distance estimation with an absolute error that is lower than 1 m and a relative error that is lower than 1.5%, which can be a reliable backup for communication-based VC operations.

## 1. Introduction

**Figure 1:**
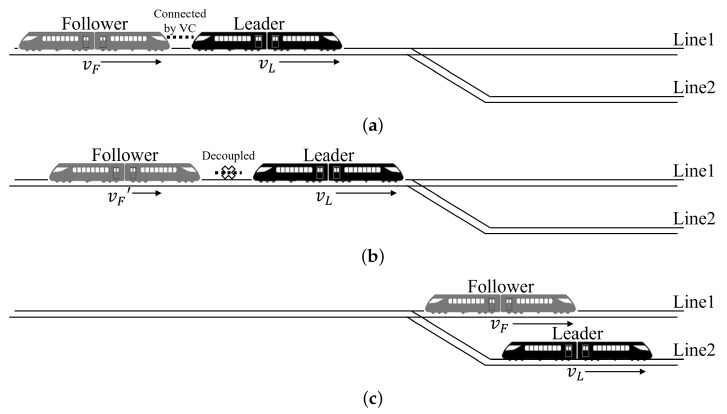
A cross-line operation scenario of VC. (**a**) A coupled VC. (**b**) A VC that is decoupled into two separated train sets before a switch. (**c**) Two decoupled train sets run separately into two lines after a switch.

Virtual coupling (VC) is considered a promising train-control mode in urban rail transit, as it enables two train sets to form one virtual train set by precisely controlling the two trains with a very close distance in operation [1]. It makes the coupling and decoupling faster and easier, does not need artificial inference, and makes cross-line operations and branching operations more flexible [2], and therefore enhances transportation efficiency [3]. As shown in Figure 1, a VC typically consists of two train sets, which are usually referred to as the leader and the follower, based on their front and rear relationships.

During the process of coupling and decoupling of VC, one of the most essential tasks is the real-time distance measurement between the two train sets [4], because the two train sets need to modify their speeds and keep a safe distance. Traditionally, the distances and speeds of the train sets can be obtained using the Global Navigation Satellite System (GNSS) and wheel sensors [5], and the position information can be exchanged using train-to-train wireless communication [6]. However, in the case of communication malfunction, alternative means need to be employed to ensure the availability of distance keeping.

Since the leader and the follower usually run within a relatively close distance, which makes them always visible for each other, distance measurement with vision sensors becomes a suitable resolution. Furthermore, the vision sensors have different principles than the train-to-train communication, which also makes them a reliable backup solution for the scenario of a communication malfunction.

When it comes to vision sensors for distance measurement, Lidar is always considered to be the most appropriate solution. By emitting and receiving laser pulses and calculating the time-of-flight, Lidar can obtain the distances to the objects. Due to its high precision and wide range [7], Lidar is widely used in autonomous driving applications such as vehicle positioning, obstacle detection, and map generation [8,9]. However, for applications in the context of urban rail transit, such as train distance measurement, Lidars have certain drawbacks. First, Lidars only provide information on distance and reflectance, and if they are not fused together with sensors of other modals, they can hardly support tasks such as object detection and tracking. Even for cases of multi-modal sensor fusion, the accuracy of multi-sensor spatial calibration can influence the performance of object detection and distance measurement [10,11]. Second, Lidars have lower frame rates, longer scan durations and sparser reflected points compared to other sensors, such as cameras. The temporal misalignment can result in more significant errors when the on-board sensors are in motion [12]. Third, from the viewpoint of deployment in practical projects, Lidars are usually space-consuming, power-consuming, and expensive as on-board devices [13]. Additionally, Lidars are more sensitive to suspended particles in the air, such as rain, snow, haze, and dust, compared to other vision sensors [14].

Apart from Lidar, a camera is also a considerable vision sensor for distance estimation in VC operation. Although cameras cannot directly acquire distances as Lidars do, they can estimate the distance by means of geometric algorithms. Differently from the active measurement of Lidars, which involves emitting laser pulses, cameras passively capture the light from objects’ surfaces, and thus information on the color, texture, and light intensity of targets can be reliably acquired [15]. Though images captured by cameras can be influenced by variations in environmental illumination, the modern high dynamic range (HDR) can make them robust to complicated illumination scenarios.

Traditionally, object distance estimation by cameras is mostly achieved by using stereo vision, in which the depth of objects is estimated by triangulation of the feature points of two stereo images [16]. For distance-estimation applications over short distances, stereo vision performs well. However, the accuracy of stereo distance estimation decreases significantly for scenarios of far distances [17]. In the context of urban rail transit, the longest distance between the two cameras of stereo vision cannot exceed the width of the train head, and while the train distance for measurement can be more than 100 m, the distance estimation can hardly be accurate. Moreover, for scenarios that contain repeated textures or textureless objects, the distance estimation can also be more unreliable [18].

To avoid the abovementioned issues of stereo vision, some researchers also explored solutions based on monocular vision [19,20]. Differently from stereo vision, monocular distance estimation utilizes information from one camera, and the distance from the camera to the object is estimated by calculating the scale factor from object sizes, shapes, perspective relationships, and the camera’s intrinsics [21]. Because of the lack of scale in monocular vision, an accurate distance estimation usually requires a priori object information and camera calibration, and it is therefore suitable for distance estimation in structural applications. In the urban rail transit context, ref. [22] proposes a monocular distance estimation method for human beings and cars on the railway track area, where the objects are detected and positioned with bounding boxes in the input images, and the distances are estimated by a well-designed neural network. Though this method is straightforward and easy to be deployed, the accuracy of distance estimation is not stable, since the detected bounding boxes cannot accurately describe the exact contours and the detailed geometric features of the objects. Ref. [23] also proposes a train distance-estimation method based on monocular vision, the distances to the train in the turnout area are estimated by instance segmentation and geometric analysis. Though this method performs well in most scenarios, since only the train side window is considered as a geometric feature, its accuracy is susceptible to image noise and bad illumination.

**Figure 2:**
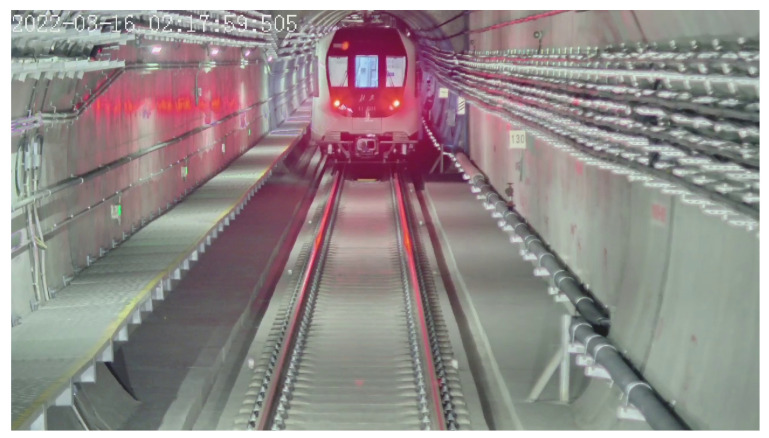
A typical image captured by an on-board camera in a virtual coupling train.

For VC scenarios in urban rail transit, the leader and the follower always run with a head-to-tail mode within visible distance, and the on-board camera in the driver cabin of one train can always capture the image of the train head of the other train, as shown in Figure 2. Considering the front windscreen, the head lights, the tail lights, and the LED screen always stay bright and have fixed positions, they can serve as key structure features used to calculate the scale of the train and thus achieve monocular distance estimation. Based on the abovementioned consideration, this paper proposes a train distance-estimation framework for virtual coupling scenarios. The main contributions of this paper are as follows:

(1) The proposed framework estimates the distance to the target train based on monocular vision, and therefore it can be a reliable backup for current VC operation, which obtains the train distance by train-to-train wireless communication.

(2) The proposed framework improves the performance of small object detection by using an additional layer in the feature pyramid network, labeling of the object neighbor area, and a semantic filter.

(3) The proposed framework improves the accuracy of the train distance estimation by using multiple key structure features in the distance estimation.

The remainder of this paper is structured as follows: Section 2 introduces the proposed framework of train distance estimation. Section 3 presents the experimental setup, implementation, results, and discussion. Finally, the paper is concluded in Section 4.

## 2. Methods

### 2.1. Framework Overview

**Figure 3:**
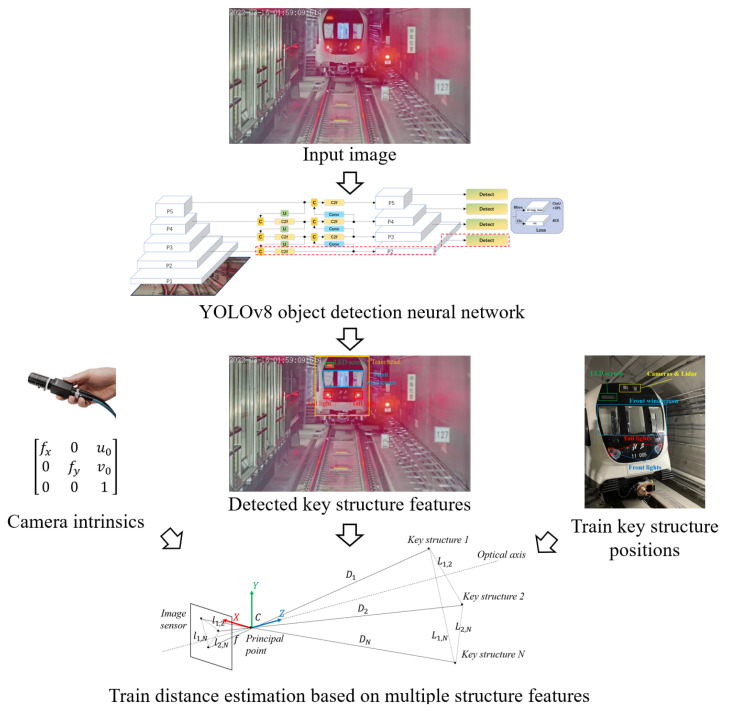
Framework overview.

In this paper, we propose a train distance-estimation framework for VC operation based on monocular vision, as shown in Figure 3. First, the key structures of the train head are extracted from the input image by a YOLOv8 object-detection neural network. To enhance the capability of small object detection, we optimized the network structure, labeling strategy, and additional semantic filter. Then, with the calibrated camera intrinsic parameters and the ground truth of key structure positions, the pixel distances between the key structure features are utilized to estimate the train distance using a pinhole camera modal.

### 2.2. Key Structure Extraction Based on Object Detection

Monocular vision utilizes the scale factor for distance estimation. Since the layout of the train head is known and stable for a given line of urban rail transit, some distances between certain key structure features can be utilized for scale factor estimation. To decrease the error as much as possible, we select the structure features that are larger in pixel dimensions and easier for detection, such as the front windscreen, front lights, tail lights, and LED screen. These structures are located at the edge of the train head and create distances with a larger scale, and since these structures are self-luminous, they can be stably detected even under poor illumination conditions, which ensures the generalization for practical applications.

**Figure 4:**
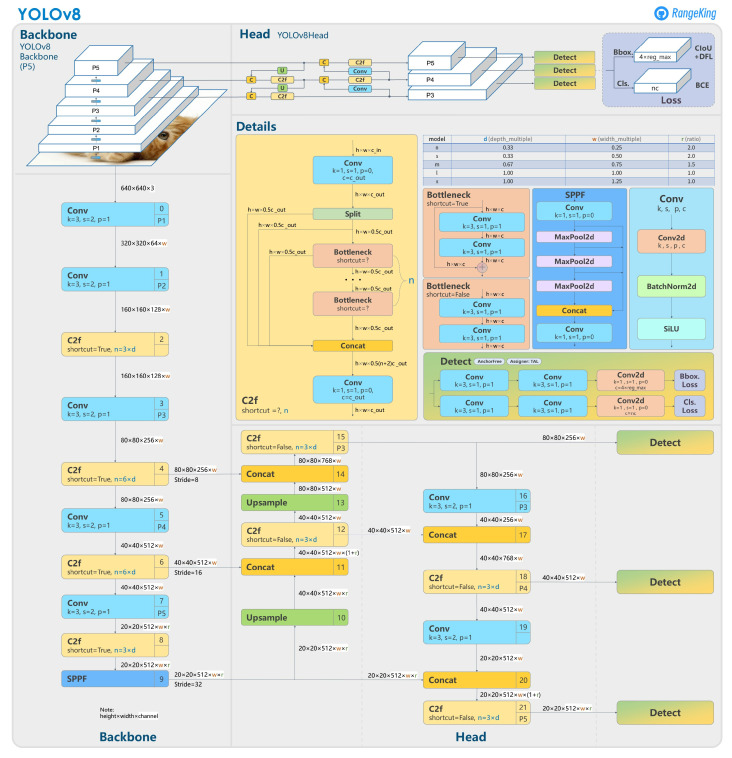
Structure of the YOLOv8 network.

To detect and locate the key structure features from the input images, object detection is the most suitable strategy. For a 2D detection task in a given visible-light RGB image, bounding boxes of rectangular shape can be used to label the positions and boundaries of the target objects and to train the neural network, and therefore predict the positions and boundaries of the detected objects. Currently, YOLO (You Only Look Once) is considered the most popular neural network series for real-time computer vision applications [24]. As a cutting-edge, state-of-the-art (SOTA) model, YOLOv8 builds on the success of previous versions, introducing new features and improvements for enhanced performance, flexibility, and efficiency [25]. YOLOv8 supports a full range of vision AI tasks, including detection, segmentation, pose estimation, tracking, and classification. This versatility allows users to leverage YOLOv8’s capabilities across diverse applications and domains. The main structure of the YOLOv8 network [26] is as shown in Figure 4.

In the YOLOv8 network, the basic features of input images are first extracted by a backbone of five convolution layers with different scales, i.e., P1, P2, P3, P4, and P5. Then, a neck of feature pyramids is utilized to concatenate the features from the outputs of P3, P4, and P5. Finally, the detection results are given by the three detection heads with different scales.

### 2.3. Optimization of Small Object Detection

**Figure 5:**
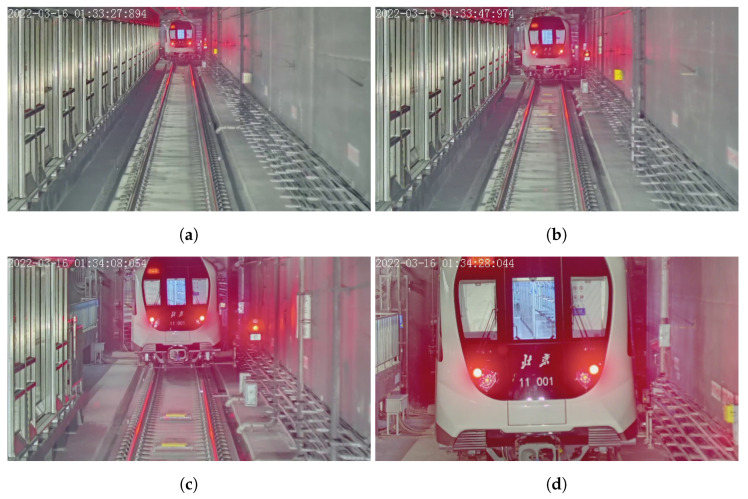
Typical images of train head captured by a long-focal-length camera at different distances. (**a**) Train head image at 113 m. (**b**) Train head image at 72 m. (**c**) Train head image at 39 m. (**d**) Train head image at 17 m.

In most VC scenarios, the distance between the leader and the follower ranges from several meters to several hundred meters, and therefore the pixel dimensions of the key structures on the train head in the captured image range from several pixels to several hundred pixels, as shown in Figure 5.

Even though the YOLO network performs well in most object-detection tasks, to ensure a stable and reliable detection of key structures with small dimensions, we propose three approaches to improve the effectiveness of small object detection: first, the object-detection neural network needs to cover more scales in the feature pyramid; second, a larger object neighbor area needs to be included in the labeling phase to provide more features for detection; and third, the semantic information of the train head can be used to exclude false-positive cases of small object detection.

#### 2.3.1. Additional Layer in the Feature Pyramid Network

**Figure 6:**
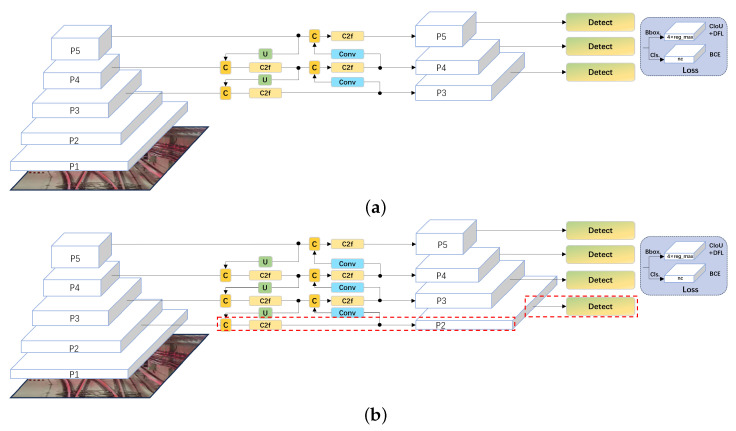
Difference of the feature pyramid network between the original YOLOv8 network and the modified YOLOv8 network. (**a**) Original YOLOv8 network. (**b**) YOLOv8 network with additional detection head of P2 layer in the feature pyramid.

In scenarios where the targets are small in input images, the original version of the YOLOv8 object detection network exhibits a significant decline in recognition performance. This is because in the original YOLOv8 network, even though the five convolution layers P1, P2, P3, P4, and P5 are utilized to extract basic features at different scales, only the outputs of P3, P4, and P5 are connected to the subsequent feature pyramid, and smaller-scale information can be lost. Therefore, this paper considers the strategy of adding more layers to the feature pyramid in order to achieve more reliable detection for small objects. The main idea is to add a P2 detection head in its direct connection, as shown in Figure 6, thus making the network more sensitive to objects with smaller pixel dimensions.

#### 2.3.2. Labeling of Object Neighbor Area

**Figure 7:**
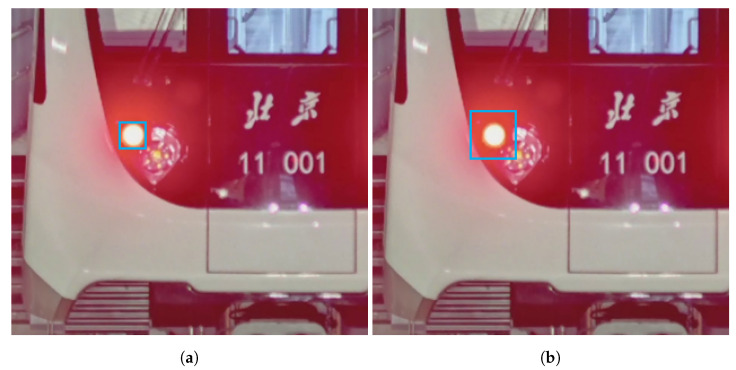
Labels with bounding boxes of different sizes (shown with blue rectangles). (**a**) A bounding box that precisely fits the size of the object. (**b**) A bounding box that is larger than the size of the object.

Some structures in the train head, such as the front lights and tail lights, have small dimensions and lack texture. Therefore, to provide more features for the network training and improve the detection recall, we made the label-bounding boxes larger than their contours, as shown in Figure 7, so as to import additional neighborhood information. While this approach may result in a decrease in the precision of object positioning, it ensures a higher sensitivity in detection, which is more important in safety-critical applications.

#### 2.3.3. Semantic Filtering

**Figure 8:**
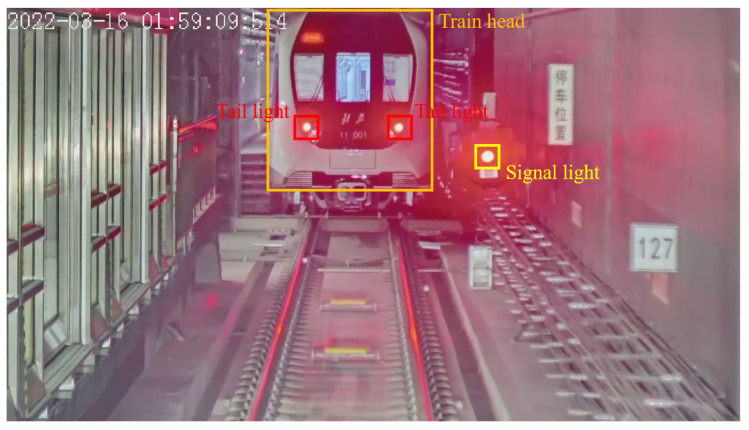
A typical scenario of false-positive, in which a signal light (shown with a yellow bounding box) was detected as a tail light. The false-positive can be filtered by the region of the train head (shown with a blue bounding box).

For small key structures, false positives are more likely to occur in the detection process. For instance, the railway signal light can easily cause confusion with the train tail light, as shown in Figure 8. Therefore, in this paper, we also train the neural network to detect the train head, and utilize the detected train head bounding box as a semantic filter to exclude the false-positive cases that are not located within the train head region.

### 2.4. Distance Estimation Based on Multiple-Feature Scale

**Figure 9:**
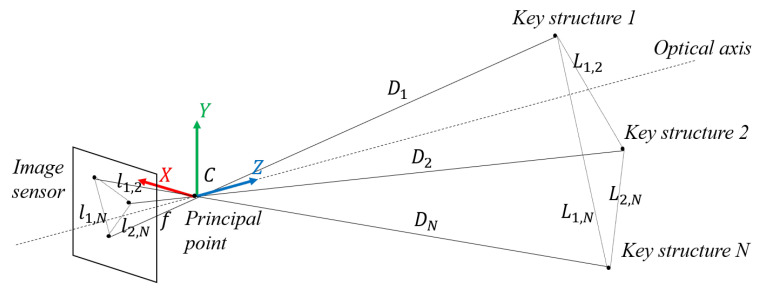
Distance estimation based on multiple structure features.

As shown in Figure 9, since the two train sets in VC are far enough from each other, and the key structure features on the train head can be considered to be on the same plane, which is perpendicular to the optical axis, in this paper, we choose the distance between every two key structure features as one restriction. For every selected two key structure features with indexes *i* and *j*, the estimated physical distance ${\hat{L}}_{i,j}$ between them can be calculated as Equation (1):

(1) $${\hat{L}}_{i,j}=\frac{l_{i,j}}{f}D_{i}$$

where $l_{i,j}$ is the pixel distance between the two key structures in the image, *f* is the focal length of the camera, and $D_{i}$ is the physical distance from the camera to key structure *i*. With the hypothesis that the structure features are far enough from the principal point of the camera and are located on the same plane, the train distances $\left\{D_{i}|i\in \left[1,N\right]\right\}$ can be considered as the same value *D*. When considering multiple features, the train distance *D* can be estimated using an optimization function calculation through weighted least squares, as shown in Equation (2):

(2) $$E=\sum_{i=1,j\neq i}^{N}w_{i,j}{\left({\hat{L}}_{i,j}-L_{i,j}\right)}^{2},$$

where $w_{i,j}$ is the weight for the distance between the *i*th and *j*th structure features, $L_{i,j}$ is the ground truth of physical distance between the two key structures, and *N* is the total number of key structure features that are taken into consideration. Note that the weight $w_{i,j}$ should be related to the physical distance, the measurement accuracy, or the appearance frequency of the feature. For example, the restriction with larger physical dimensions should be given with a larger weight in the distance estimation, since its relative error in the measurement tends to be smaller. In this paper, we use the physical distance $L_{i,j}$ as the weight.

## 3. Experiments

To validate and evaluate the proposed distance estimation framework, we implemented experiments on Beijing Subway Line 11.

### 3.1. Experiment Setup

**Figure 10:**
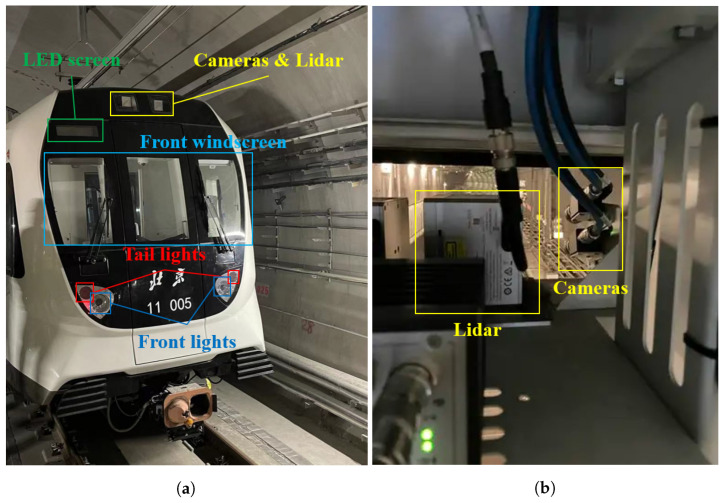
Experiment setup of train in Beijing Subway Line 11. (**a**) Outside view of the train head. (**b**) Inside view of the sensor setup.

As shown in Figure 10, a long-focal length camera and a Lidar were mounted inside the roof of the train driver cabin by means of a customized metal supporter. The used camera has a 1280 × 720 pixel resolution and 120 dB high dynamic range (HDR) capability, which enables starlight-level imaging. Equipped with a lens with 25 mm focal length, the camera offers a field of view of 16.0° in the horizontal direction and 9.0° in the vertical direction. The intrinsics of the camera were calibrated by Zhang’s camera-calibration method in advance [27], and the results are as shown in Table 1, where $f_{x}$ and $f_{y}$ are the focal lengths along the two orthogonal directions, and $u_{0}$ and $v_{0}$ are the coordinates of the principal point in the image reference frame. The Lidar used in this study is a Livox Tele-15 [13] with a field view angle of 14.5° × 16.2°. To obtain a better forward view, the camera’s pitch angle was set to 8°, while the Lidar was mounted horizontally.

### 3.2. Dataset

**Figure 11:**
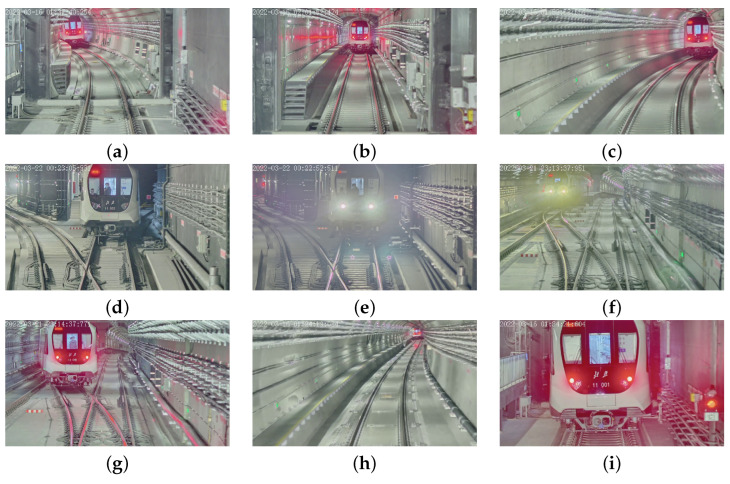
Representative images of the dataset. (**a**) Left curve. (**b**) Straight line. (**c**) Right curve. (**d**) With personals in the driver cabin. (**e**) Front light on. (**f**) Front light with different color. (**g**) Train in adjacent railway. (**h**) Train at a very far distance (more than 200 m). (**i**) Train at a very close distance (less than 30 m).

To train the object-detection neural network, we collected and labeled 555 images from the on-board camera of Beijing Subway Line 11 to build the dataset. Some of representative images in the dataset are as shown in Figure 11, and they come from many typical scenarios. The dataset was separated into a training set, a validation set, and a test set with a proportion of 6:2:2. In each input image, five types of key structures were labeled with bounding boxes, which include the train head, LED screen, front windscreen, tail light, and head light.

### 3.3. Small Object Detection

To study how the proposed approaches influence the performance of small object detection, in this paper, we take the train tail light as the object, since its small size, round shape, and lack of texture will likely lead to false positives or false negatives.

In this paper, to obtain a balance between precision and model volume, we chose the YOLOv8m as the base model, which supports an input of 640 pixels and 25.9 M parameters (78.9 FLOPs). To study the effectiveness of adding more layers of detection heads to the detection network, we added a P2 layer to the YOLOv8m model as the YOLOv8m-p2 model. Additionally, to evaluate the effectiveness of the labeling of object neighbor areas, we enlarged the bounding box sizes of the original dataset to 2.5 times the original sizes by a step length of 0.1, while fixing their center positions, and thus obtained a total of 16 datasets.

Then, the YOLOv8m model and YOLOv8m-p2 model were both trained with the 16 datasets using an Nvidia RTX3070 GPU. Considering the size of the models, we configured the training with a batch size of 8 for 500 epochs. All trainings used the same configuration, as shown in Table 2.

In order to make better use of the dataset, we also implemented data augmentation during the training process. A number of parameters such as HSV (hue, saturation, value) adjustments, the translation range, the scale range, etc., were modified for data augmentation, and the details are as shown in Table 3.

To evaluate the trained models quantitatively, we utilized two widely used indicators, precision and recall. The calculation formulas are as follows:

(3) $$Precision=\frac{TP}{TP+FP}$$

(4) $$Recall=\frac{TP}{TP+FN}$$

where TP refers to true positive; FP refers to false positive; and FN refers to false negative. Since we also worried about the influence of enlarging the bounding box on the positional precision of the object detection, we also implemented corresponding experiments, in which the positional errors of detected objects were analyzed by root mean square error (RMSE) with a unit of one pixel.

**Figure 12:**
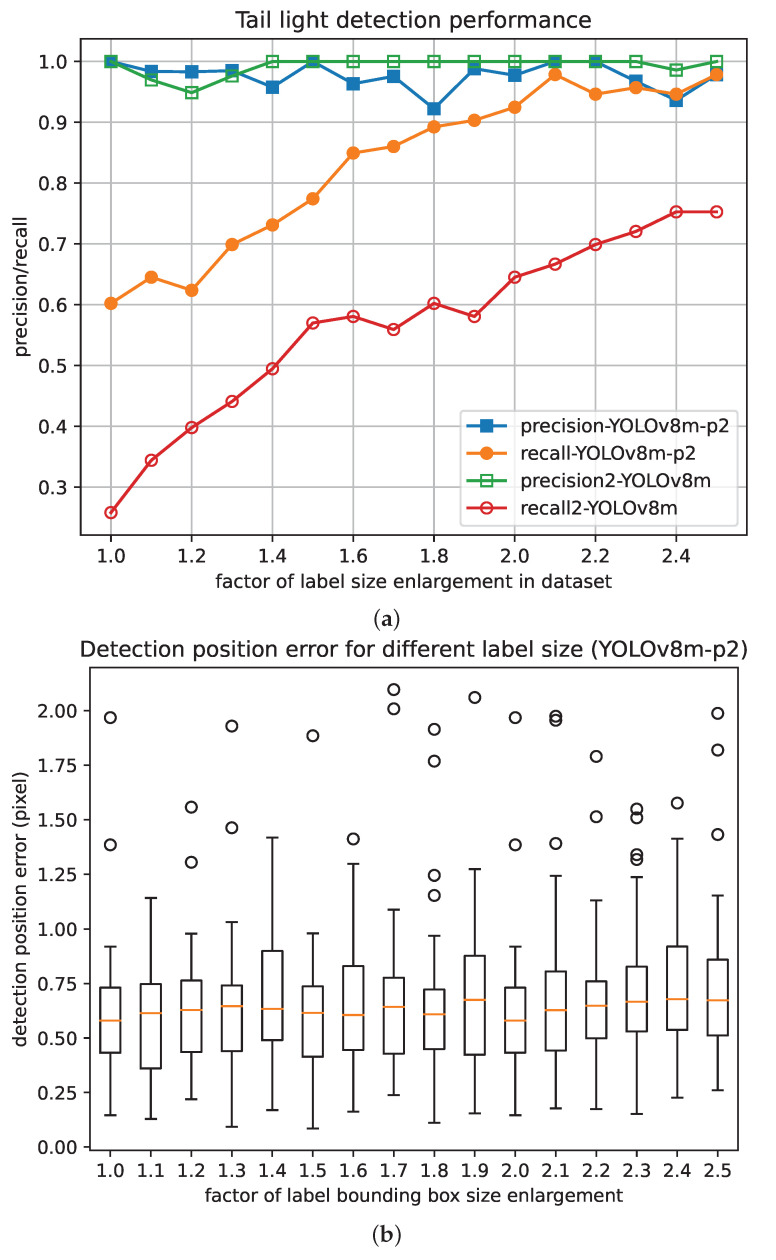
Detection performance with different labeling bounding box enlargements. (**a**) Detection recall and precision of train tail light. (**b**) Positioning error.

As shown in Figure 12, while YOLOv8m-p2 performed slightly worse than YOLOv8m regarding precision, the recall of YOLOv8m-p2 was significantly higher than that of YOLOv8m. Since the reliability of the distance-estimation function in VC is the priority, and the semantic filtering can exclude most of false positive cases, YOLOv8m-p2 is more acceptable than YOLOv8m. Additionally, for both YOLOv8m-p2 and YOLOv8m, as the labeling bounding boxes were enlarged from the original sizes two-fold, the recall increased significantly while the precision did not show a significant change.

For the positional precision of object detection, when the labeled areas were enlarged from original sizes to 2.5 times the original sizes, though the detection precision showed a slight fluctuation, the recall increased significantly while the position precision did not show a significant change.

### 3.4. Distance Estimation

To quantitatively evaluate the distance estimation performance of the proposed framework, we designed experiments of distance estimation under different train distance conditions.

**Figure 13:**
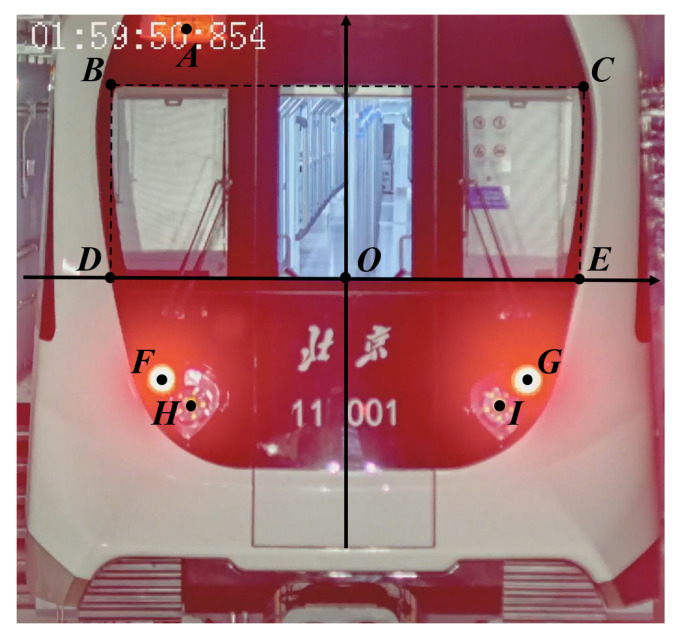
Key structures for distance estimation. The positions of the 9 key structures are signed by point *A* to point *I* in the image.

In this paper, we chose the LED screen, front windscreen, tail lights and front lights as key structures in the train head for distance estimation, as shown in Figure 13. The relative positions of the key structures are as shown in Table 4.

**Figure 14:**
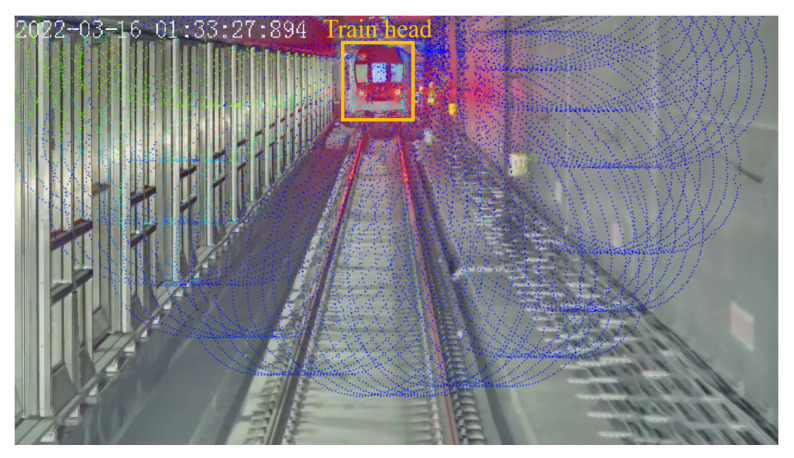
Ground truth by projecting Lidar point clouds onto camera image (the blue and green dots represent the projected point cloud).

For reference, the data of on-board Lidar is taken as the ground truth to calculate the distance estimation errors. The extrinsics between the camera and the Lidar are calibrated first, and the point clouds are projected onto the detected train head bounding box in the camera image, as shown in Figure 14. The position of the point cloud that is located near the center of the two tail lights is selected as the ground truth position of the train head.

**Figure 15:**
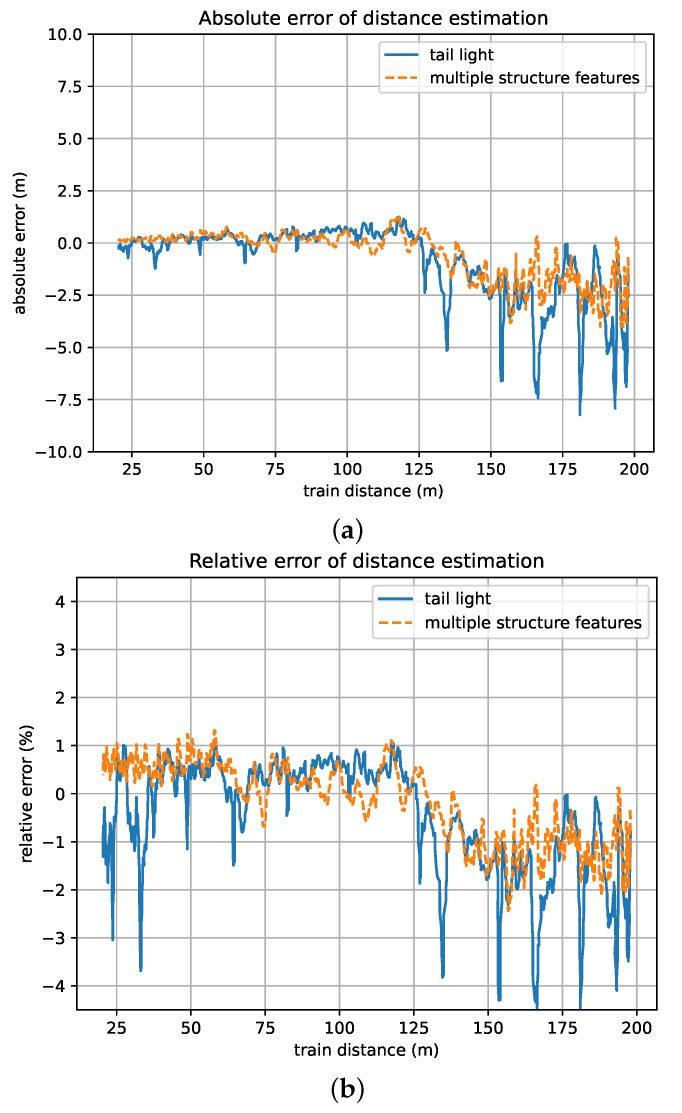
Errors of the proposed framework under different train distance conditions. (**a**) Absolute distance error. (**b**) Relative distance error.

Considering the most common scenarios of VC, we chose a typical case in which the follower of the VC moves toward a stationary leader from distances ranging from 200 m to 20 m to evaluate the precision of distance estimation under different train distance conditions. In addition, to validate the effectiveness of the optimization method based on multiple structure features, in the experiments, we first chose the tail lights in the train head as the only key structure feature and then chose the tail lights, LED screen, and front windscreen as key structure features. The results are as shown in Figure 15.

According to the results, the distance estimation error that resulted by using multiple key structure features fluctuates in a smaller range than the one that resulted by only using the tail light as the key structure feature. The maximal resulting errors under different train distances can be seen in Table 5.

### 3.5. Discussion

According to the experimental results, the proposed framework provided a satisfactory accuracy of train distance estimation in VC operation scenarios. In the phase of the key structure feature extraction, by adding a detection head in P2 layer of feature pyramid network in the YOLOv8 neural network, the recall for small objects improved significantly. Additionally, by enlarging the label bounding boxes for small objects, the recall was further improved without a loss of detection position precision. When the size of the label bounding box was set to be more than 2.0 times that of the object contour, the recall increased to more than 95%, which is an acceptable level for practical application. In the phase of train distance estimation, when using multiple key structure features in the distance estimation calculation, the distance error showed less fluctuations than when only using one kind of key structure feature. For scenarios in which the train distance ranged from 20 m to 100 m, the absolute error of the distance estimation remained lower than 1 m. While the distance estimation precision of the proposed framework may not match that of a Lidar, it is still sufficient for train distance control in VC backup mode. Moreover, the proposed framework requires only one camera, which simplifies the hardware structure and eliminates the need for complex procedures such as multi-sensor time–space synchronization.

## 4. Conclusions

In this paper, a train distance estimation framework based on monocular vision is proposed for the backup of virtual coupling operations. Multiple structure features of the target train are extracted by an object-detection neural network and employed to estimate the train distance. For validation and evaluation, we implemented experiments on Beijing Subway Line 11, and a dataset that includes more than 500 images was built for key structure feature extraction. According to the experimental results, by adding a P2 detection head to the feature pyramid of the YOLOv8 neural network, labeling the object neighbor area, and filtering false positives using semantic information of the train head, the performance of small object detection can be improved. The experimental results of distance estimation show that the proposed framework can estimate the train distance with an absolute error of less than 1 m and a relative error of less than 1.5% for scenarios where the train distance ranges from 20 m to 100 m, and is a considerable backup for virtual coupling operations.

However, this paper also has limitations. First, in this paper, we only utilized an object detection approach for key structure feature extraction. Future work will also explore the possibility of pose detection for key structure feature extraction. Second, this paper only discussed the straight line distance between the two train sets of VC, and future work will also explore the influence of railway track curvature on train distance estimation. Third, limited to the data source, in this paper we only studied the tunnel scenario of urban rail transit. Future work will also explore the train distance estimation solution for outdoor scenarios, in which the illumination changes could be more challenging.

## Figures and Tables

**Table 1:** Intrinsic parameters of on-board camera.

Parameter	Value
$f_{x}$	4562.66
$f_{y}$	4557.50
$u_{0}$	640.55
$v_{0}$	383.86

**Table 2:** Training configuration.

Parameter	Value	Parameter	Value
Epochs	500	Patience	50
Batch	8	Image Size	640
Save	True	Save Period	−1
Device	Auto	Workers	8
Project	Null	Name	Null
Pretrained	True	Optimizer	Auto
Verbose	True	Seed	0
Deterministic	True	Cosine LR	False
Mixed Precision	True	Validation	True
Validation Split	Val	Conf. Threshold	Null
IoU Threshold	0.7	Max Detection	300
Overlap Mask	True	Mask Ratio	4
Dropout	0.0	Workspace	4
nms	False	lr0	0.01
lrf	0.01	Momentum	0.937
Weight Decay	0.0005	Warm-Up Epochs	3.0
Warm-Up Momentum	0.8	Warm-Up Bias lr	0.1
Box	7.5	cls	0.5
dfl	1.5	Pose	12.0
kobj	1.0	Label Smoothing	0.0
nbs	64		

**Table 3:** Data augmentation configuration.

Parameter	Value	Parameter	Value
HSV Hue Range	0.015	HSV Saturation Range	0.7
HSV Value Range	0.4	Degrees Range	0.0
Translation Range	0.1	Scale Range	0.5
Shear Range	0.0	Perspective Range	0.0
Vertical Flip Prob.	0.0	Horizontal Flip Prob.	0.5
Mosaic Augmentation	1.0		

**Table 4:** Key structure relative positions.

Point	Key Structure Name	Position Coordinate (m)
A	LED screen center	[−0.69, 1.08]
B	front windscreen (upper left)	[−1.02, 0.83]
C	front windscreen (upper right)	[1.02, 0.83]
D	front windscreen (lower left)	[−1.02, 0]
E	front windscreen (lower right)	[1.02, 0]
F	tail light center (left)	[−0.79, −0.45]
G	tail light center (right)	[0.79, −0.45]
H	front light center (left)	[−0.67, −0.56]
I	front light center (right)	[0.67, −0.56]

**Table 5:** Maximums of train distance estimation errors.

Distance Range	Absolute Error (Tail Lights)	Absolute Error (Multiple Structures)	Relative Error (Tail Lights)	Relative error (Multiple Structures)
20–50 m	1.22 m	0.61 m	3.69%	1.23%
50–100 m	0.96 m	0.76 m	1.49%	1.31%
100–200 m	8.23 m	4.00 m	4.54%	2.44%

## Data Availability

The data utilized in this study are proprietary and confidential data held by the company Traffic Control Technology Co., Ltd., and are protected by confidentiality agreements and legal regulations. Due to the sensitive and confidential nature of the data, they are not publicly accessible. For further research interests or access requests, please contact the data administrator or relevant department to obtain additional information and permissions.
